# The potential utility of evoked potentials in the treatment of mental illnesses

**DOI:** 10.1093/psyrad/kkad024

**Published:** 2023-10-26

**Authors:** Salvatore Campanella

**Affiliations:** Laboratoire de Psychologie Médicale et d'Addictologie, ULB Neuroscience Institute (UNI), CHU Brugmann-Université Libre de Bruxelles (U.L.B.), 1020 Brussels, Belgium

The “Decade of the Brain” referred to the 1990s, as the emergence of various brain imaging techniques allowed the analysis of both normal and pathological behaviors with specific patterns of distributed neural activity (Insel and Quirion, 2005). Among these techniques, electroencephalography (EEG) indexed spontaneous brain electrical activity, and included event-related potentials (ERPs), referring to epochs of EEG activity that are time-locked to the processing of stimuli (Rugg and Coles, 1995). Once mental diseases were also envisaged as resulting from brain alterations (Price *et al*., 2000), a neurocognitive approach emerged, promoting that: (i) significant cognitive disturbances are observed in psychiatric diseases (Green, 2006); (ii) these cognitive disturbances, indexed by dysfunctional neural networks, may trigger/subtend the onset and/or maintenance of clinical symptoms, thereby defining valid therapeutic targets (Verdejo-Garcia *et al*., 2023); and (iii) rehabilitating these cognitive functions (through neuropsychological training programs and/or neuromodulation tools) disclosed encouraging results by promoting reduction of clinical symptoms as well as enhancement of patients’ quality of life (Lesniak *et al*., 2014). In this view, ERPs, considered a useful tool to probe the information processing stream in the brain, can help pinpoint the specific neurocognitive functions that should be targeted in each patient through specific and individualized cognitive remediation procedures (Campanella, 2016). Obviously, even if we choose to focus this perspective paper on cognitive ERPs, several other electrophysiological tools (such as transcranial magnetic stimulation or neural oscillations) have proved to be useful for studying neurophysiological biomarkers of psychiatric disorders (Cao *et al*., 2021; Ferrarelli and Phillips, 2021).

Focusing on cognitive ERPs was triggered by the current idea that it is important to evaluate which cognitive dysfunction(s) may subtend the onset and/or maintenance of a clinical symptom to be able to rehabilitate (them) and to reduce the severity of this clinical symptom. Indeed, empirical evidence indicates that (i) some cognitive ERP markers can predict the clinical trajectory of psychiatric patients (Dousset *et al*., 2022; Kim *et al*., 2023); and (ii) cognitive training programs combined with neuromodulation tools can induce specific neural changes (Campanella *et al*., 2017; Schroder *et al*., 2020; Dousset *et al*., 2021) and promote clinical symptom reduction (Monnart *et al*., 2019; Dubuson *et al*., 2021). The most interesting part of such findings was to suggest that cognitive disturbances are closely linked to the onset and maintenance of clinical symptoms (e.g. altered inhibitory skill can favor negative intrusive thoughts in depressive disorders as well as a relapse in alcohol dependence; Monnart *et al*., 2016; Petit *et al*., 2014), or that various ERP parameters, i.e. the oddball P300 and the No-Go P300 components (ERP waveforms classically recorded thanks to the oddball and the Go/No-go tasks), could predict abstinence vs. relapse at 3 months in recently detoxified alcoholic patients (Campanella *et al*., 2020).

Indexing cognitive alterations could of course be done through neuropsychological testing, but this is too time consuming in psychiatric settings. Cognitive ERPs may then be used to quickly target main cognitive restrictions. However, despite being non-invasive, globally available, and cost-effective, and showing decades of research with recent encouraging results, the clinical utility of ERPs in psychiatry is still poor. A theoretically grounded framework to concretely apply ERPs in psychiatric care units has been proposed (Campanella, 2021). Currently, clinical symptoms are at the first plan to drive psychiatric evaluations, and the clinical trajectory is monitored by clinical interviews. However, reaching a diagnostic does not usually include the use of biomarkers (Casey *et al*., 2013). Once the idea that disturbed brain networks also subtend psychiatric diseases was admitted, a further assumption was to acknowledge that these neural alterations should be reflected in long-lasting neural modifications to trigger enduring real-world behavioral changes (Vinogradov *et al*., 2012). Therefore, we promoted test–retest ERP sessions at the individual level to favor “individualized” or “personalized” medicine (Campanella *et al*., 2019). Indeed, ERP markers may help (i) to monitor the spontaneous and/or treatment-related evolution of the specific neurocognitive processes that triggered the onset and persistence of all clinical symptoms observed in singular patients; and (ii) to evaluate whether these brain modifications triggered by the treatment can predict the clinical evolution of the patient (Fig. 1). Two recent ERP case reports showed, for instance, a perfect congruency between the clinical and the neurophysiological evolution (indexed through the P300 component) of a psychotic patient (Kajosch *et al*., 2020), and that cognitive ERPs may be used as relevant indicators of cognitive vulnerabilities in individual patients (Ingels *et al*., 2022).

**Figure 1:**
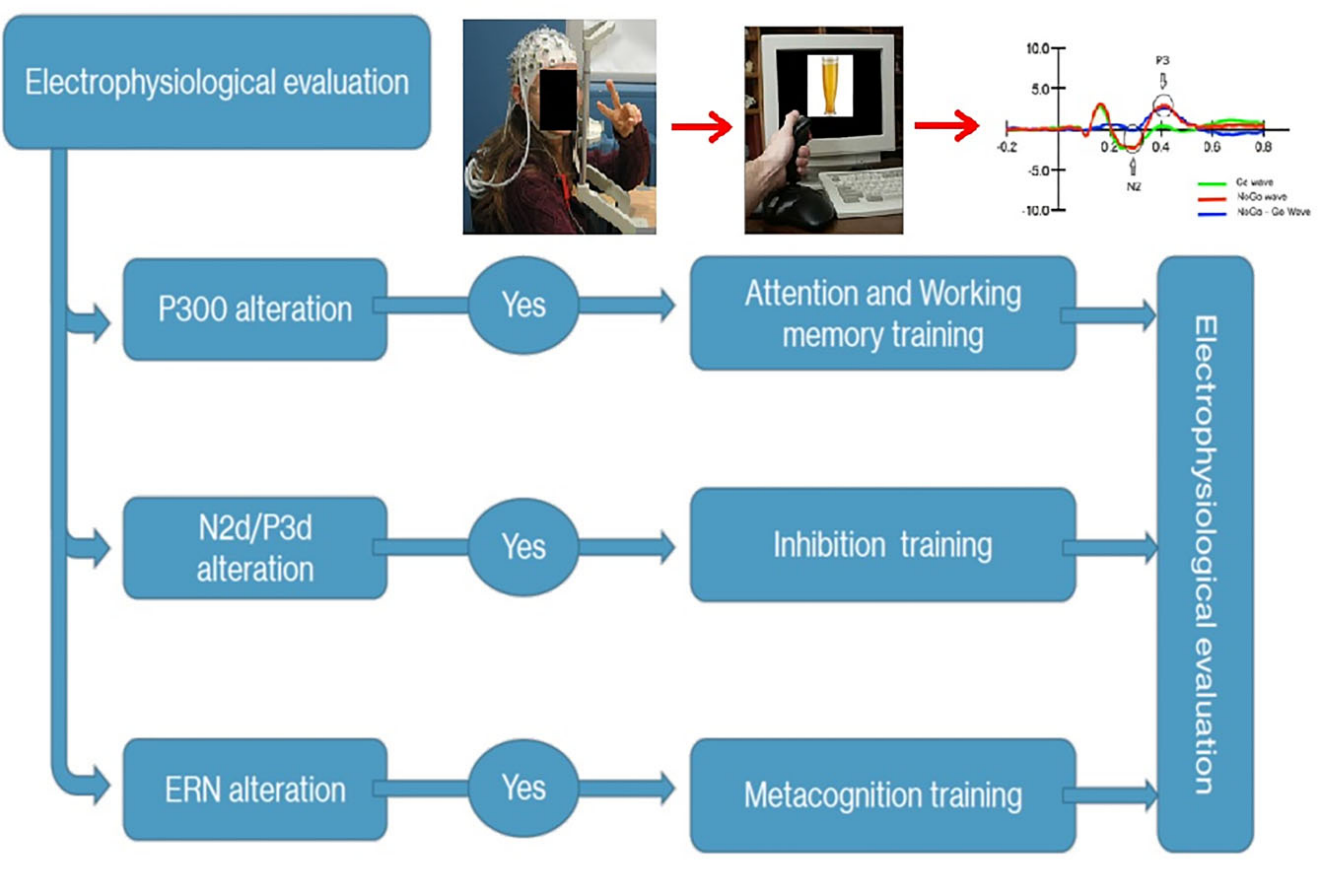
Illustration of ERP screening on an individual patient through test–retest sessions to orient neurocognitive rehabilitation.

To reach this aim, three main directions have been proposed (Campanella, 2021): (i) the definition of clear and applicable multi-site guidelines to validate an unambiguous set of normative data; (ii) the promotion of a multi-component ERP approach; and (iii) the development of ERP serial recordings.

First, at the methodological level, the worldwide current ERP literature is represented by a huge number of ERP studies reporting several conflicting results, mainly due to technical variations (Hajcak *et al*., 2019). Such variability in reported data has of course generated a lot of scepticism among clinicians as it raises questions regarding reliability. At the clinical level, technical guidelines already exist for some main ERPs (e.g. the P300, Duncan *et al*., 2009); however, their use is still by no means widespread throughout the world, and this could sometimes lead to some misunderstanding of the data (Campanella and Colin, 2014). Developing such normative technical guides adopted by the whole field would clearly help with clinical implementation. In this way, we would guarantee access to normative data recorded from large samples using similar procedures so as to index the progression of patients’ scores as a function of the treatment, but also ensuring that the impact of some potential confounding variables (sex, age, drug, or comorbidity for instance) was controlled for.

Second, a main advantage of ERPs is to present a high degree of sensitivity combined with some predictive power. However, it suffers from poor specificity (Pogarell, 2007). Accordingly, it could be argued that a “multivariate endophenotype,” mainly based on a weighted mixture of various ERP components (P50, P300, and mismatch negativity), may help to furnish a reliable diagnostic more accurately than any single component (Price *et al*., 2006). Further ERP recordings should then use a multi-component approach to potentially increase the sensitivity and/or specificity of ERP studies by decreasing the impact of group heterogeneity. This could also allow a better specification of the individual cognitive process(es) that should be reeducated in a singular patient.

Third, ERPs represented a well-suited tool for using serial recordings in follow-up studies to index neural modifications induced by a specific treatment (Pogarell *et al*., 2007). Indeed, if there are differences in waveshape, size, and timing of ERPs between individuals, ERPs have disclosed high stability within an individual due to a high internal consistency and a high test–retest reliability (Kappenman *et al*., 2017). Consequently, as ERPs can be recorded many times from the same individual with high reliability, session-related changes reported in brain activity should result from treatment intervention or disease evolution in a singular patient (Kappenman and Luck, 2016). Overall, by fostering a multi-component follow-up approach, ERPs may be used to monitor and/or predict specific modifications in brain function in response to therapy (e.g. psycho-social support, medication) as well as to index specific remaining cognitive alterations. In this way, clinicians may promote adapted individual interventions that will be tailored to the specific needs of an individual patient, thus providing a “personalized” medicine.

## References

[bib1] Campanella S (2016) Neurocognitive rehabilitation for addiction medicine: from neurophysiological markers to cognitive rehabilitation and relapse prevention. Prog Brain Res. 224:85–103. 26822355 10.1016/bs.pbr.2015.07.014

[bib2] Campanella S (2021) Use of cognitive event-related potentials in the management of psychiatric disorders: towards an individual follow-up and multi-component clinical approach. WJP. 11:153. 34046312 10.5498/wjp.v11.i5.153 PMC8134870

[bib3] Campanella S, Colin CC (2014) Event-related potentials and biomarkers of psychiatric diseases: the necessity to adopt and develop multi-site guidelines. Front Behav Neurosci. 8:428. 25540614 10.3389/fnbeh.2014.00428 PMC4261801

[bib4] Campanella S, Schroder E, Kajosch H et al. (2019) Why cognitive event-related potentials (ERPs) should have a role in the management of alcohol disorders. Neurosci Biobehav Rev. 106:234–44. 29936112 10.1016/j.neubiorev.2018.06.016

[bib5] Campanella S, Schroder E, Kajosch H et al. (2020) Neurophysiological markers of cue reactivity and inhibition subtend a three-month period of complete alcohol abstinence. Clin Neurophysiol. 131:555–65. 31786051 10.1016/j.clinph.2019.10.020

[bib6] Campanella S, Schroder E, Monnart A et al. (2017) Transcranial direct current stimulation over the right frontal inferior cortex decreases neural activity needed to achieve inhibition: a double-blind ERP study in a male population. Clin EEG Neurosci. 48:176–88. 27170671 10.1177/1550059416645977

[bib7] Cao K-X, Ma M-L, Wang C-Z et al. (2021) TMS-EEG: an emerging tool to study the neurophysiologic biomarkers of psychiatric disorders. Neuropharmacology. 197:108574. 33894219 10.1016/j.neuropharm.2021.108574

[bib8] Casey BJ, Craddock N, Cuthbert BN et al. (2013) DSM-5 and RDoC: progress in psychiatry research?. Nat Rev Neurosci. 14:810–4. 24135697 10.1038/nrn3621 PMC4372467

[bib9] Dousset C, Ingels A, Schröder E et al. (2021) Transcranial direct current stimulation combined with cognitive training induces response inhibition facilitation through distinct neural responses according to the stimulation site: a follow-up event-related potentials study. Clin EEG Neurosci. 52:181–92. 32924586 10.1177/1550059420958967

[bib10] Dousset C, Schroder E, Ingels A et al. (2022) Intact error-related negativity at the start of a three-weeks detoxification program reflects a short-term protective factor against relapse in alcoholic patients: some preliminary evidence from a follow-up event-related potentials study. Clin EEG Neurosci. 53:316–25. 35125020 10.1177/15500594221076579

[bib11] Dubuson M, Kornreich C, Vanderhasselt M-A et al. (2021) Transcranial direct current stimulation combined with alcohol cue inhibitory control training reduces the risk of early alcohol relapse: a randomized placebo-controlled clinical trial. Brain Stimulation. 14:1531–43. 34687964 10.1016/j.brs.2021.10.386

[bib12] Duncan CC, Barry RJ, Connolly JF et al. (2009) Event-related potentials in clinical research: guidelines for eliciting, recording, and quantifying mismatch negativity, P300, and N400. Clin Neurophysiol. 120:1883–908. 19796989 10.1016/j.clinph.2009.07.045

[bib13] Ferrarelli F, Phillips ML (2021) Examining and modulating neural circuits in psychiatric disorders with transcranial magnetic stimulation and electroencephalography: present practices and future developments. AJP. 178:400–13. 10.1176/appi.ajp.2020.20071050 PMC8119323 33653120

[bib14] Green MF (2006) Cognitive impairment and functional outcome in schizophrenia and bipolar disorder. J Clin Psychiatry. 67:3. 17107235

[bib15] Hajcak G, Klawohn J, Meyer A (2019) The utility of event-related potentials in clinical psychology. Annu. Rev. Clin. Psychol. 15:71–95. 31067414 10.1146/annurev-clinpsy-050718-095457

[bib16] Ingels A, Fabry L, Hanak C et al. (2022) Using cognitive event-related potentials in the management of alcohol use disorder: towards an individual approach. Arch Clin Med Case Rep. 06:772–5.

[bib17] Insel TR (2005) Psychiatry as a clinical neuroscience discipline. JAMA. 294:2221–4. 16264165 10.1001/jama.294.17.2221 PMC1586100

[bib18] Kajosch H, Hanard F, Steegen G et al. (2020) Monitoring the clinical evolution of a psychotic patient presenting a first-schizophrenic episode thanks to bimodal oddball-P300 event-related potentials: first evidence from a single-case study. Arch Clin Med Case Rep. 04:1194–207.

[bib19] Kappenman ES, Keil A (2017) Introduction to the special issue on recentering science: replication, robustness, and reproducibility in psychophysiology. Psychophysiology. 54:3–5. 28000258 10.1111/psyp.12787

[bib20] Kappenman ES, Luck SJ (2016) Best practices for event-related potential research in clinical populations. Biol. Psychiatry Cogn Neurosci Neuroimaging. 1:110–5. 27004261 10.1016/j.bpsc.2015.11.007 PMC4797328

[bib21] Kim M, Kim T, Hwang WJ et al. (2023) Forecasting prognostic trajectories with mismatch negativity in early psychosis. Psychol Med. 53:1489–99. 36315242 10.1017/S0033291721003068 PMC10009395

[bib22] Leśniak M, Polanowska K, Seniów J, Członkowska A (2014) Effects of repeated anodal tDCS coupled with cognitive training for patients with severe traumatic brain injury: a pilot randomized controlled trial. J Head Trauma Rehabil. 29:E20–9. 23756431 10.1097/HTR.0b013e318292a4c2

[bib23] Monnart A, Kornreich C, Verbanck P, Campanella S (2016) Just swap out of negative vibes? Rumination and inhibition deficits in major depressive disorder: data from event-related potentials studies. Front. Psychol. 7:1019. 27516743 10.3389/fpsyg.2016.01019 PMC4963408

[bib24] Monnart A, Vanderhasselt M-A, Schroder E et al. (2019) Treatment of resistant depression: a pilot study assessing the efficacy of a tDCS-mindfulness program compared with a tDCS-relaxation program. Front. Psychiatry. 10:730. 31708808 10.3389/fpsyt.2019.00730 PMC6819945

[bib25] Petit G, Cimochowska A, Kornreich C et al. (2014) Neurophysiological correlates of response inhibition predict relapse in detoxified alcoholic patients: some preliminary evidence from event-related potentials. Neuropsychiatr Dis Treat. 10:1025. 24966675 10.2147/NDT.S61475 PMC4062548

[bib26] Pogarell O, Mulert C, Hegerl U (2007) Event-related potentials in psychiatry. Clin EEG Neurosci. 38:25–34. 17319589 10.1177/155005940703800108

[bib27] Price BH, Adams RD, Coyle JT (2000) Neurology and psychiatry: closing the great divide. Neurology. 54:8–8. 10636118 10.1212/wnl.54.1.8

[bib28] Price GW, Michie PT, Johnston J et al. (2006) A multivariate electrophysiological endophenotype, from a unitary cohort, shows greater research utility than any single feature in the Western Australian family study of schizophrenia. Biol Psychiatry. 60:1–10. 16368076 10.1016/j.biopsych.2005.09.010

[bib29] Rugg MD, Coles MG (1995) Electrophysiology of Mind: Event-Related Brain Potentials and Cognition. Oxford University Press. https://global.oup.com/academic/product/electrophysiology-of-mind-9780198524168?cc=be&lang=en&

[bib30] Schroder E, Dubuson M, Dousset C et al. (2020) Training inhibitory control induced robust neural changes when behavior is affected: a follow-up study using cognitive event-related potentials. Clin EEG Neurosci. 51:303–16. 31858835 10.1177/1550059419895146

[bib31] Verdejo‐Garcia A, Rezapour T, Giddens E et al. (2023) Cognitive training and remediation interventions for substance use disorders: a Delphi consensus study. Addiction. 118:935–51. 36508168 10.1111/add.16109 PMC10073279

[bib32] Vinogradov S, Fisher M, De Villers-Sidani E (2012) Cognitive training for impaired neural systems in neuropsychiatric illness. Neuropsychopharmacol. 37:43–76. 10.1038/npp.2011.251 PMC3238091 22048465

